# Circulating trace elements status in COVID-19 disease: A meta-analysis

**DOI:** 10.3389/fnut.2022.982032

**Published:** 2022-08-12

**Authors:** Yunhui Li, Weihe Luo, Bin Liang

**Affiliations:** ^1^Clinical Laboratory, PLA North Military Command Region General Hospital, Shenyang, China; ^2^Department of Medical Engineering, PLA North Military Command Region General Hospital, Shenyang, China; ^3^Department of Bioinformatics, Key Laboratory of Cell Biology, Ministry of Public Health, and Key Laboratory of Medical Cell Biology, Ministry of Education, School of Life Sciences, China Medical University, Shenyang, China

**Keywords:** SARS-CoV-2, COVID-19, trace element, serum, prognosis, meta-analysis

## Abstract

Trace elements are a group of essential metals or metalloids, which are necessary for life, and present in minute amounts. Despite substantial researches highlighting the importance of trace elements in Coronavirus disease 2019 (COVID-19) diseases, a thorough evaluation of the levels of circulating trace elements is lacking. Therefore, we conducted a systematic review and meta-analysis to evaluate the trace element status (Zn, Fe, Cu, Mg, and Se) in COVID-19 disease. We also assessed the relationship between circulating trace elements and COVID-19 disease severity and survival status during follow-up. We searched comprehensively MEDLINE, Web of Science, CNKI, and WangFang databases without language restriction, between November 1, 2019 and April 1, 2022. The search identified 1,566 preliminary references. A total of 49 studies met the eligibility criteria and were included in the review, and 42 studies were included in the final meta-analysis. Meta-analysis showed that COVID-19 patients had significantly lower circulating Zn (SMD: −0.83, 95% CI: −1.19 to −0.46, *P* < 0.001), Fe (SMD: −1.56, 95% CI: −2.90 to −0.21, *P* = 0.023), and Se (SMD: −0.75, 95% CI: −0.94 to −0.56, *P* < 0.001) levels than healthy controls, and circulating Zn (SMD: −0.47, 95% CI: −0.75 to −0.18, *P* = 0.002), Fe (SMD: −0.45, 95% CI: −0.79 to −0.12, *P* = 0.008), and Se (SMD: −0.27, 95% CI: −0.49 to −0.04, *P* = 0.020) levels were associated with the presence of severity status in COVID-19 patients. Moreover, circulating Fe levels in non-survivors were significantly lower than survivors in COVID-19 (SMD: −0.28, 95% CI: −0.44 to −0.12, *P* = 0.001). However, there was no significant difference in Cu and Mg levels between COVID-19 patients and controls, severity and non-severity status, and survivors and non-survivors (all *P* > 0.05). Taken together, COVID-19 patients displayed lower circulating levels of Zn, Fe, and Se, and their levels were associated with severity status. Moreover, circulating Fe levels may provide part of the explanation for the unfavorable survival status. Therefore, we presumed optimistically that supplements of trace elements might provide an adjutant treatment in the early stages of COVID-19.

**Systematic review registration:**

[https://www.crd.york.ac.uk/prospero], identifier [CRD42022348599].

## Introduction

The global Coronavirus disease 2019 (COVID-19), caused by Severe Acute Respiratory Syndrome Coronavirus-2 (SARS-CoV-2), has becoming a pandemic disease in March, 2020 (1). Based on the World Health Organization (WHO) declaration, as of May13, 2022, there have been 517,648,631 confirmed cases of COVID-19, including 6,261,708 deaths (2). COVID-19 presents with a broad clinical spectrum, ranging from mild fever to fatigue, cough, severe pneumonia, acute respiratory distress syndrome (ARDS), affecting the kidneys, brain, liver, gastrointestinal tract, heart, and other organs (1, 3, 4). The present studies indicated that the mortality varies according to sex, age, disease severity, circumstance, and patient comorbidities (mainly chronic lung diseases, hypertension, diabetes, and coronary heart disease) (5, 6). In view of the rapid spread and much more contagiousness of SARS-CoV-2 variants (7), the COVID-19 pandemic has posed a serious threat to the lives and health of people around the world.

Trace elements are a group of essential metals or metalloids, which are necessary for life, and present in minute amounts (8). Trace elements are involved in various biological, chemical and molecular processes, which regulates cellular homeostasis, humoral and cellular immune responses and acts as cofactors for many enzymes and antioxidant molecules (9). It has been established that zinc (Zn), iron (Fe), copper (Cu), magnesium (Mg), and selenium (Se) play vital and synergistic roles at every stage of the immune response (10). Deficiency of trace elements could affect innate and adaptive immune response, which predisposes to infections and further aggravates malnutrition (11). At present, there are also a number of studies and reviews highlighting a potentially important roles of trace elements in the pathogenesis of COVID-19, and investigating the possibility of utilization of trace elements in diagnosis, prognosis and supplements in therapeutic procedures (12–14).

Zn is an essential trace element which has a variety of fundamental biological functions, such as antioxidant, anti-inflammatory, and apoptotic effects (15–17). Zn element also involved in DNA synthesis, cellular integrity, cell division, cell proliferation, cell differentiation, and cell signal transduction as a second messenger (18). Besides being essential for a fundamental biological functions, the present study indicated that Zn deficiency could weaken the ability of the human body toward SARS-CoV-2 infection and increases the risk of overactive immune response to cause tissue damage(19). Moreover, Ivanova ID et al. found that Zn and Cu levels were abnormal dynamically during the course of COVID-19, and were mainly associated with the inflammation response (20). Based on previous literature, it is clear that maintaining optimum levels of Zn and Cu may stimulate both innate and adaptive immune systems in the course of viral infection. Another important metal element, Fe, is a key functional components for many proteins and enzymes involved in vital cellular processes, and a vital nutrient affecting immunity (21, 22). Previous studies suggested that Fe deficiency was associated with decreased immunity to pathogens and poorer response to some vaccines (23, 24). Domenico Girelli et al. indicated that severe COVID-19 appears to be characterized by marked functional Fe deficiency, which was possibly related to impaired immune response (21). Mg is an essential nutrient required for many different metabolic and biochemical function. In view of the importance of Mg in maintaining proper immune, vascular and pulmonary function, Valentina Trapani et al. had proposed that Mg homeostasis could affect the susceptibility and the response to SARS-CoV-2 (25). Subsequent preclinical works showed that Mg does have protective effects against COVID-19 infection, and derangement in Mg homeostasis might contribute to and aggravate COVID-19 syndrome (26–28). To date, accumulating evidence has indicating that Se play a role in anti-inflammatory, antiviral, and oxidative stress and immune-cell activity, and is a prerequisite for proper immune system functioning (29–33). The recent researches suggested that a deficiency of Se may decrease the immune defenses against COVID-19 and is associated with COVID-19 disease severity and mortality (34–36). Based on these experimental and clinical data, Zn, Cu, Fe, Mg, and Se were considered as the important trace elements, which involved in the process of COVID-19.

Despite substantial researches highlighting the importance of trace elements in COVID-19 disease, a thorough evaluation of the levels of circulating trace elements is lacking. Due to a variety of populations, different measuring methods, different reference values, geographic features, and dietary habits, previous studies have shown the conflicting results on the association of circulating trace elements and COVID-19. Therefore, it is hard to extrapolate a univocal conclusion from the existing evidence. The overall objective of the present study was to evaluate the trace element status in COVID-19 disease by conducting a meta-analysis. We also assessed the relationships between circulating trace elements and COVID-19 disease severity and survival status during follow-up.

## Materials and methods

### Search strategy

The systematic review and meta-analysis were conducted following a recently published protocol (37), and reported according to the guidelines of the PRISMA (Preferred Reporting Items for Systematic Reviews and Meta-Analysis)(38). We searched comprehensively MEDLINE, Web of Science, CNKI, and WangFang databases without language restriction, between 1st November 2019 and 1st April 2022. In literature search, we used MeSH terms: (COVID-19[MeSH Terms]) AND (trace elements [MeSH Terms]). The related key words were used for all of the databases: (“trace elements” OR “zinc” OR “iron” OR “copper” OR “magnesium” OR “selenium”) AND (“COVID-19” or “SARS-CoV-2” OR “coronavirus”) AND (“blood” OR “serum” OR “plasma” OR ‘circulating”). In addition, the reference lists of the primary studies were evaluated and screened to find other relevant studies.

### Selection criteria

In order to investigate the difference between blood trace element levels in COVID-19 patients and controls, and associations between blood trace element levels and COVID-19 severity and survival status, we used the inclusion and exclusion criteria to identify relevant articles. The inclusion criteria were as follows: (a) A diagnosed COVID-19 disease, (b) reporting blood levels of trace elements (Zn, Cu, Fe, Mg, and Se) in COVID-19 patients and matched controls, (c) COVID-19 patients with different degree of disease severity or survival status. The detailed COVID-19 severity classification criteria used in the evaluation of original studies and meta-analyses was based on international guidelines or Acute Physiology and Chronic Health Evaluation. Severe and critical categories were defined as severe, mild and moderate as non-severe in data analysis. The exclusion criteria were: (a) studies with incomplete data; (b) pregnant COVID-19 cases and pediatric cases; (c) case reports, letters, reviews, comment, and animal studies; (d) duplicate publication. All literature was independently reviewed by two authors (LB and LYH). Any discrepancy was solved through discussion.

### Data extraction and quality assessment

The extract data included first author, publication year, country, sample size, study design, ages of COVID-19 patients and controls, sex, disease severity, and survival outcome. Two authors independently collected the data, and finally reached an agreement. The quality of included studies were assessed using Newcastle-Ottawa Scale (NOS).

### Statistical analysis

STATA 14.0 software (STATA Corp., College Station, TX, United States) was used to analyze the available data. The standard mean difference (SMD) with 95% confidence intervals (CIs) was estimated the difference of circulating trace element levels between COVID-19 patients and controls, severe COVID-19 infection and non-severe COVID-19 infection, survivors and non-survivors. Assessment of heterogeneity was performed using Cochran’s Q statistics (*P* < 0.1) and *I*^2^ statistics (*I*^2^ > 50%), in which a *P* < 0.10 or *I*^2^ > 50% indicated significant heterogeneity. A random-effect model was adopted to calculate the pooled SMD and 95% CI in the presence of significant heterogeneity, otherwise, a fixed-effect model was conducted. Sensitivity analyses were performed to evaluate the influence of each study on the overall effect size using the leave-one-out method. The funnel plots, Egger’s test, and Begg’s test were used to evaluate publication bias. A *P* < 0.05 was considered statistically significant.

## Results

### Identification of included studies

**FIGURE 1:**
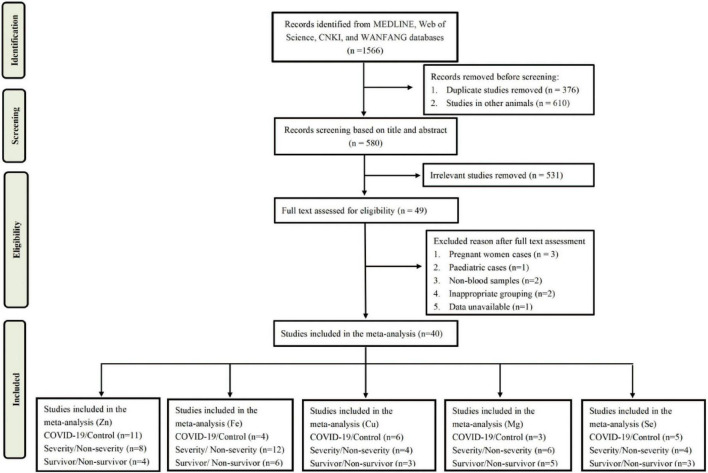
Flow diagram of the study selection process.

A total of 1,566 articles were obtained from MEDLINE, Web of Science, CNKI, and WanFang databases. After screening, 49 articles were selected for eligibility in the analysis. After reviewing the full text, 9 articles were excluded due to non-blood samples, inappropriate grouping, data unavailable, and inappropriate samples (pregnant cases and children cases). Finally, there were 40 studies met the inclusion and exclusion criteria in the meta-analysis. A flow chart of the literature search was shown in Figure 1.

### Characteristics of included studies

The characteristics of all included studies were present in Tables 1–3, respectively. These studies were published ranging from 2020 to 2022. Of 40 studies, 18 articles reported Zn levels (11 articles: COVID-19 vs. controls; 8 articles: severity vs. non-severity; 4 articles: survivor vs. non-survivors)(12, 20, 27, 39–53), 15 articles reported Fe levels (4 articles: COVID-19 vs. controls; 12 articles: severity vs. non-severity; 6 articles: survivor vs. non-survivors) (12, 27, 40, 51, 54–64), 9 articles reported Cu levels (5 articles: COVID-19 vs. controls; 4 articles: severity vs. non-severity; 3 articles: survivor vs. non-survivors) (12, 20, 27, 39, 47, 49–51, 65), 10 articles reported Mg levels (3 articles: COVID-19 vs. controls; 5 articles: severity vs. non-severity; 6 articles: survivor vs. non-survivors)(27, 28, 40, 50, 51, 66–70), and 9 articles reported Se levels (5 articles: COVID-19 vs. controls; 4 articles: severity vs. non-severity; 3 articles: survivor vs. non-survivors) (34, 36, 39, 44, 47, 49, 51, 71, 72). Overall, the studies were performed in 16 countries across Asia, Europe, America and Oceania. Moreover, it is noteworthy that 3,735 participants (1,336 COVID-19 cases and 2,399 controls, Table 1), 2,076 participants (818 severe COVID-19 cases and 1,258 non-severe COVID-19 cases, Table 2), and 3,248 participants (2,677 survivors and 571 non-survivors, Table 3) were included in the present analysis, respectively. The samples types in all included studies were serum or plasma or whole blood. The risk of bias assessment based on NOS was displayed in Supplementary Table 1.

**TABLE 1:** The main characteristics of included studies in comparison of trace elements between COVID-19 cases and controls.

References	Country	Study design	COVOD-19/control	Cases (Male/female)	Case (Age)	Controls (male/female)	Control (Age)	Sample type	Elements
Al-Jassas et al. (66)	Iraq	Case-control	60/30	NA	48.6 ± 8.5	NA	47.1 ± 5.2	Serum	Mg
Bastin et al. (54)	Iran	Cross-sectional	147/39	87/60	NA	18/21	NA	Serum	Fe
Elham et al. (41)	Iran	Case-control	93/186	41/52	51(40–61)	NA	NA	Serum	Zn
Ghanei et al. (42)	Iran	Case-control	90/95	35/55	52 ± 16	33/62	48 ± 19	Serum	Zn
Golabi et al. (43)	Iran	Cross-sectional	53/53	36/32	41 ± 13	38/34	40 ± 14	Serum	Zn
Ivanova et al. (20)	Bulgaria	Cohort	75/68	39/36	62.5 ± 14.91	32/36	53.7 ± 12.84	Serum	Zn, Cu
Jothimani et al. (45)	India	Cohort	47/45	29/18	34(18–77)	30/15	32.0(18–60)	Serum	Zn
Kocak et al. (47)	Turkey	Case-control	60/32	32/28	Asymptomatic: 41.25(24–25); mild: 31.9(16–72); moderate:54.1(23–96); severe:58(39–85)	11/21	45.5(21–82)	Serum	Zn, Cu, Se
Maares et al. (48)	Germany	Cross-sectional	33/86	19/14	Male: 81(65–82); female: 82(75–89)	57/29	male:34(26–47); female: 35(24–42)	Serum	Zn
Majeed et al. (36)	India	Case-control	30/30	24/6	40.5(37.5–43)	14/16	33.5(26–37)	Serum	Se
Nedic et al. (12)	Serbia	Case-control	60/20	31/29	51.4 ± 12.6	9/11	54.1 ± 14.3	Serum	Zn, Fe, Cu
Pvsn et al. (50)	India	Cohort	150/50	101/49	Mild: 60.91 ± 10.96; moderate:59.26 ± 11.77; severe:57.04 ± 13.2	23/27	30.8 ± 8.11	Serum	Zn, Cu, Mg
Skalny et al. (51)	Russia	Cohort	150/43	81/69	Mild: 50.47 ± 15.91; moderate:54.22 ± ± 12.5; severe:64.5 ± 15.49	27/16	55.67 ± 4.36	Serum	Zn, Fe, Cu, Mg, Se
Skesters et al. (71)	Latvia	Cohort	40/40	NA	NA	NA	NA	Plasma	Se
Verschelden et al. (52)	Belgium	Cohort	139/1513	91/48	65(54–77)	1513	NA	Plasma	Zn
Yagci et al. (63)	Turkey	Cross-sectional	59/19	54/24	mild:60.89 ± 12.409; severe:64.11 ± 10.718; critical:65.59 ± 11.253	13/6	63.47 ± 11.467	Serum	Fe
Younesian et al. (72)	Iran	Cross-sectional	50/50	NA	77.8 ± 13.9	NA	91.7 ± 16.7	Serum	Se

NA,not available.

**TABLE 2:** The main characteristics of included studies in comparison of trace elements between severity status and non-severity status in COVID-19 patients.

References	Country	Study design	Reported groups	Groups in meta- analysis	Numbers (S/non-S)	Severity (Male/female)	Severity (Age)	Non-severity (male/female)	Non-severity (Age)	Sample type	Elements
Al-Saleh et al. (39)	Saudi Arabia	Cohort	Mild/ moderate/ severe	S/non-S	89/15	11/4	69(67–81)	49/40	69(62–77.5)	Serum	Zn, Cu, Se
Bastin et al. (54)	Iran	Cross-sectional	Mild/ moderate/ severe/ critical	S/non-S	170/56	29/27	NA	NA	NA	Serum	Fe
Beigmohammadi et al. (40)	Iran	Cross-sectional	Apache score ≥ 25/ Apache score < 25	S/non-S	27/6	2/4	NA	12/15	NA	Serum	Zn, Fe, Mg
Chakurkar et al. (56)	India	Cohort	Mild/ moderate/ severe	S/non-S	165/14	11/3	66.79 ± 17.51	97/68	54.30 ± 15.78	Serum	Fe
Razeghi Jahromi et al. (44)	Iran	Cohort	Mild/ moderate/ severe	S/non-S	37/13	10/3	72(65–77)	21/16	49(42–66)	Serum	Zn, Se
Kilercik et al. (57)	Germany	Cohort	Mild +moderate/ severe +critical	S/non-S	27/6	2/4	89(81–94)	12/15	69(38–91)	Serum	Fe
Kocak et al. (47)	Turkey	Case-control	Mild/ moderate/ severe	S/non-S	46/24	15/13	61.0 ± 15.8	24/18	60.9 ± 15	Serum	Zn, Cu, Se
Lanser et al. (58)	Austria	Retrospective cohort	Mild +moderate/ severe	S/non-S	68/15	10/5	73(47–95)	34/34	63(31–89)	Serum	Fe
Lv et al. (59)	China	Retrospective cohort	Non-severe/ severe	S/non-S	547/82	NA	NA	NA	NA	Serum	Fe
Pvsn et al. (50)	India	Cohort	Mild/ moderate/ severe	S/non-S	396/63	49/14	70.58 ± 10.66	271/125	60.39 ± 11.47	Serum	Zn, Cu, Mg
Quilliot et al. (69)	France	Prospective cohort	Moderate/ severe/ critical	S/non-S	36/23	NA	NA	NA	NA	Serum	Mg
Skalny et al. (51)	Russia	Cohort	Mild/ moderate/ severe	S/non-S	261/101	62/39	79 ± 12	53/208	72 ± 16	Serum	Zn, Fe, Cu, Mg, Se
Sonnweber et al. (61)	Austria	Cohort	Mild/ moderate/ severe	S/non-S	99/21	13/8	60.0(51.0–74.0)	NA	NA	Serum	Fe
Tojo et al. (62)	Japan	Retrospective/ prospective cohort	Non-RF/mild-RF/severe-RF	S/non-S	549/96	NA	80(75–88)	NA	NA	Serum	Fe
Yagci et al. (63)	Turkey	Cross-sectional	Mild/ severe/ critical	S/non-S	43/7	NA	NA	NA	NA	Serum	Fe
Yasui et al. (53)	Japan	Cohort	Mild +moderate/ severe	S/non-S	89/22	14/8	64.4(59.3–74.9)	56/33	55.5(48.3–63.3)	Serum	Zn
Zeng et al. (27)	China	Retrospective cohort	Non-severe/ severe	S/non-S	28/7	2/5	89(81–94)	13/15	69(38–91)	Whole blood	Zn, Fe, Cu, Mg
Zhao et al. (64)	China	Retrospective cohort	Mild/ severe/ critical	S/non-S	89/15	11/4	69(67–81)	49/40	69(62–77.5)	Serum	Fe
Zhu et al. (70)	China	Retrospective cohort	Moderate/ severe/ critical	S/non-S	170/56	29/27	NA	NA	NA	Serum	Mg

S/Non-S: severity/non-severity; NA, not available.

**TABLE 3:** The main characteristics of included studies in comparison of trace elements between survival and non-survival in COVID-19 patients.

References	Country	Study design	Reported groups	Groups in meta-analysis	Number (survival/non-survival)	Non-survivor (male/female)	Non-survivor (Age)	Survivor (male/female)	Survivor (Age)	Sample type	Elements
Zeng et al. (27)	China	Retrospective cohort	Recovered/ Deceased	Survival/ Non-survival	89/15	11/4	69(67–81)	49/40	69(62–77.5)	Whole blood	Zn, Fe, Cu, Mg
Bagher Pour et al. (49)	Iran	Prospective cohort	Recovered/ Deceased	Survival/ Non-survival	170/56	29/27	NA	NA	NA	Serum	Zn, Cu, Se
Maares et al. (48)	Germany	Cross-sectional	Discharge/ Death	Survival/ Non-survival	27/6	2/4	NA	12/15	NA	Serum	Zn
Joulaei et al. (46)	Iran	Cross-sectional	Survivor/ Non-survivor	Survival/ Non-survival	165/14	11/3	66.79 ± 17.51	97/68	54.30 ± 15.78	Serum	Zn
Younesian et al. (72)	Iran	Cross-sectional	Survivor/ Non-survivor	Survival/ Non-survival	37/13	10/3	72(65–77)	21/16	49(42–66)	Serum	Se
Moghaddam et al. (34)	Germany	Cross-sectional	Discharge/ Death	Survival/ Non-survival	27/6	2/4	89(81–94)	12/15	69(38–91)	Serum	Se
Bonakdaran et al. (67)	Iran	Cross-sectional	Survivor/ Non-survivor	Survival/ Non-survival	46/24	15/13	61.0 ± 15.8	24/18	60.9 ± 15	Serum	Mg
Zhu et al. (70)	China	Retrospective cohort	Survivor/ Non-survivor	Survival/ Non-survival	68/15	10/5	73(47–95)	34/34	63(31–89)	Serum	Mg
Gunay et al. (68)	Turkey	Retrospective cohort	Survivor/ Non-survivor	Survival/ Non-survival	547/82	NA	NA	NA	NA	Serum	Mg
Alamdari et al. (28)	Iran	Retrospective cross-sectional	Discharge/ expired	Survival/ Non-survival	396/63	49/14	70.58 ± 10.66	271/125	60.39 ± 11.47	Serum	Mg
Yagci et al. (63)	Turkey	Cross-sectional	Survivor/exitus	Survival/ Non-survival	36/23	NA	NA	NA	NA	Serum	Fe
Bianconi et al. (55)	Iran	Prospective cohort	Survivor/ Non-survivor	Survival/ Non-survival	261/101	62/39	79 ± 12	53/208	72 ± 16	Serum	Fe
Chakurkar et al. (56)	India	Prospective cohort	Alive/Death	Survival/ Non-survival	99/21	13/8	60.0(51.0–74.0)	NA	NA	Serum	Fe
Lanser et al. (58)	Austria	Retrospective cohort	Mild +moderate/ death	Survival/ Non-survival	549/96	NA	80(75–88)	NA	NA	Serum	Fe
Zhao et al. (64)	China	Retrospective cohort	Survivor/ Non-survivor	Survival/ Non-survival	43/7	NA	NA	NA	NA	Serum	Fe
Nai et al. (60)	Italy	Prospective cohort	Survivor/ Non-survivor	Survival/ Non-survival	89/22	14/8	64.4(59.3–74.9)	56/33	55.5(48.3–63.3)	Plasma	Fe
Hackler et al. (65)	Germany	Cross-sectional	Discharge/ Death	Survival/ Non-survival	28/7	2/5	89(81–94)	13/15	69(38–91)	Serum	Cu

NA, not available.

### Meta-analysis of trace elements in COVID-19 patients

#### Zn levels in COVID-19 patients

**FIGURE 2:**
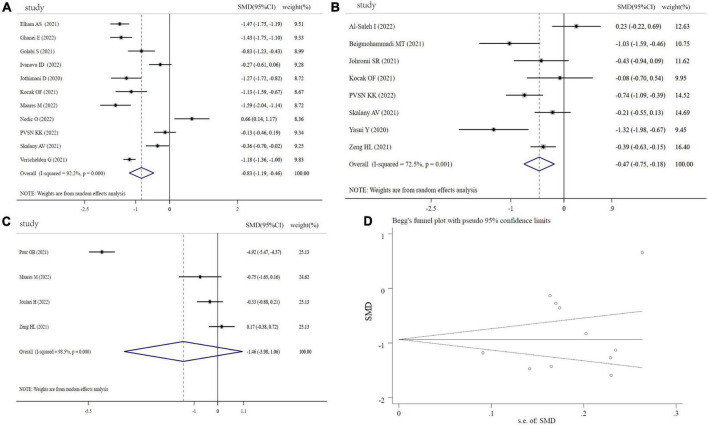
Forest plot of Zn levels. **(A)** Zn levels between COVID-19 patients and controls; **(B)** Zn levels between severity status and non-severity status in COVID-19 patients; **(C)** Zn levels between non-survivors and survivors in COVID-19 patients; **(D)** funnel plot of the meta-analysis on Zn levels between COVID-19 patients and controls.

Figures 2A–C depicts the forest plots for the pooled estimates of the SMD on Zn levels between the COVID-19 patients and controls, severity and non-severity, and non-survivors and survivors. Due to the significant heterogeneity (COVID-19 patients vs. controls: *I^2^* = 92.2%, *P* < 0.001; severity vs. non-severity: *I^2^* = 72.5%, *P* < 0.001; survivors vs. non-survivors *I^2^* = 98.5%, *P* < 0.001), a random-effect model was used. As shown in Figure 2A, combining 11 effective sizes from 11 studies showed that the overall SMD in Zn levels between the COVID-19 patients and controls was −0.83(−1.19 to 0.46, *P* < 0.001), indicating that COVID-19 patients had significantly lower Zn levels. Moreover, we evaluated the differences of Zn level between severity and non-severity, or survivors and non-survivors. The COVID-19 patients with severity status had significantly lower Zn levels than COVID-19 patients with non-severity status (SMD: −0.47, 95% CI: −0.75 to −0.18, *P* = 0.002). However, Zn levels in the non-survivors were not significantly different from the survivors in COVID-19 patients (SMD: −1.46, 95%CI: −3.98 to 1.06, *P* = 0.256). There was no significant publication bias in the analysis using Begg’s test (*P* = 0.350) and Egger’s test (*P* = 0.312, Figure 2D). As shown in Supplementary Figure 1, sensitivity analysis revealed that the exclusion of any single study had no significant impact on pooled SMD.

#### Fe levels in COVID-19 patients

**FIGURE 3:**
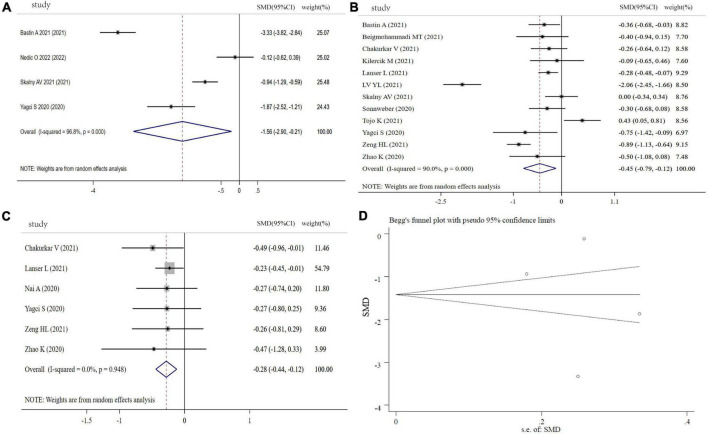
Forest plot of Fe levels. **(A)** Fe levels between COVID-19 patients and controls; **(B)** Fe levels between severity status and non-severity status in COVID-19 patients; **(C)** Fe levels between non-survivors and survivors in COVID-19 patients; **(D)** funnel plot of the meta-analysis on Fe levels between COVID-19 patients and controls.

Fe levels were analyzed between COVID-19 patients and controls (4 studies), severity and non-severity status (12 studies), and the non-survivors and survivors (6 studies) in COVID-19 patients. Compared with controls, COVID-19 patients showed a significantly lower circulating levels of Fe (SMD: −1.56, 95% CI: −2.90 to −0.21, *P* = 0.023, Heterogeneity: *I^2^* = 96.8%, *P* < 0.001), shown in Figure 3A. Figures 3B,C revealed that COVID-19 patients with severity status or non-survivors had a significant lower Fe levels than COVID patients with non-severe status or survivors (severity vs. non-severity: SMD: −0.45, 95% CI: −0.79 to −0.12, *P* = 0.008, Heterogeneity: *I^2^* = 90%, *P* < 0.001; non-survivors vs. survivors: SMD: −0.28, 95% CI: −0.44 to −0.12, *P* < 0.001, Heterogeneity: *I^2^* = 0%, *P* = 0.948). The sensitivity analysis demonstrated the stability of the pooled SMD (Supplementary Figure 2), and funnel plot, Begg’s test (*P* = 1.00) and Egger’s test (*P* = 0.711) displayed no significant publication bias (Figure 3D).

#### Cu levels in COVID-19 patients

**FIGURE 4:**
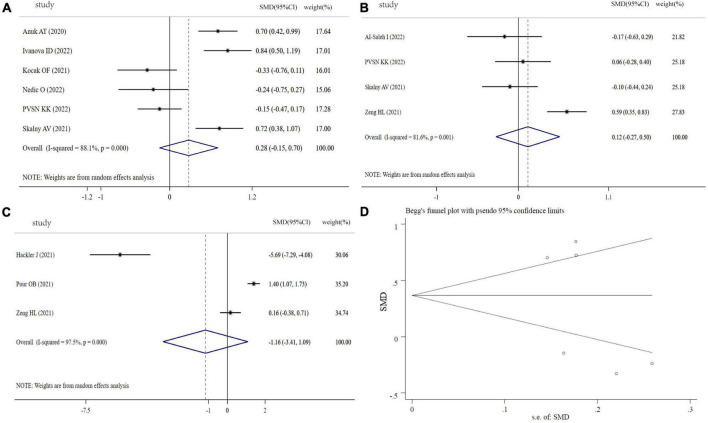
Forest plot of Cu levels. **(A)** Cu levels between COVID-19 patients and controls; **(B)** Cu levels between severity status and non-severity status in COVID-19 patients; **(C)** Cu levels between non-survivors and survivors in COVID-19 patients; **(D)** funnel plot of the meta-analysis on Cu levels between COVID-19 patients and controls.

Figure 4A shows the results of six studies comparing the circulating Cu levels between COVID-19 cases and controls. Compared with the controls, the COVID-19 patients displayed no significant difference in Cu levels (SMD: 0.28, 95% CI: −0.15 to 0.70, *P* = 0.246, heterogeneity: *I^2^* = 88.1%, *P* < 0.001). Moreover, we found that COVID−19 patients with severity status and the non-survivors had similar Cu levels in comparison with COVID-19 patients with non-severity status (SMD: 0.12, 95% CI: −0.27 to 0.50, *P* = 0.126, heterogeneity: *I^2^* = 81.6%, *P* = 0.001) and the survivors (SMD: −1.16, 95% CI: −3.41 to 1.09, *P* = 0.312, heterogeneity: *I^2^* = 97.5%, *P* < 0.001), respectively (Figures 4B,C). Sensitivity analyses generated similar results with the primary meta-analysis (Supplementary Figure 3). Based on funnel plot, Begg’s test (*P* = 0.707) and Egger’s test (*P* = 0.244), there was no publication bias in this meta-analysis (Figure 4D).

### Mg levels in COVID-19 patients

**FIGURE 5:**
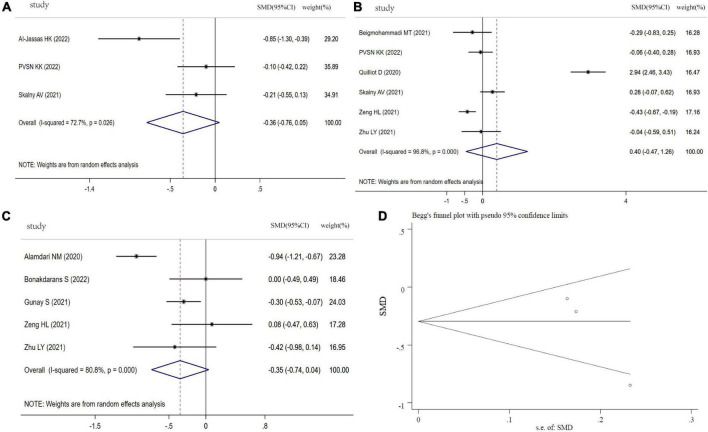
Forest plot of Mg levels. **(A)** Mg levels between COVID-19 patients and controls; **(B)** Mg levels between severity status and non-severity status in COVID-19 patients; **(C)** Mg levels between non-survivors and survivors in COVID-19 patients; **(D)** funnel plot of the meta-analysis on Mg levels between COVID-19 patients and controls.

As indicated in Figure 5A, the SMD of circulating Mg levels between COVID-19 patients and controls was pooled from 3 studies, and no significant difference was found (SMD: −0.36, 95% CI: −0.76 to 0.05, *P* = 0.08, heterogeneity: *I^2^* = 72.7%, *P* = 0.026). Figure 5B showed that there was no significant difference in COVID-19 patients between severity status and non-severity status (SMD: 0.40, 95% CI: −0.47 to 1.26, *P* = 0.30, heterogeneity: *I^2^* = 96.8%, *P* < 0.001). The COVID-19 non-survivors had the same Mg levels with survivors (SMD: −0.35, 95% CI: −0.74 to 0.04, *P* = 0.079, heterogeneity: *I^2^* = 80.8%, *P* < 0.001), as shown in Figure 5C. The sensitivity analysis suggested that the result is not due to the effect of any single study (Supplementary Figure 4). Visual inspection of funnel plot symmetry suggested a significant potential publication bias, examined by Begg’s test (*P* = 0.296) and Egger’s test (*P* = 0.004) (Figure 5D).

#### Se levels in COVID-19 patients

**FIGURE 6:**
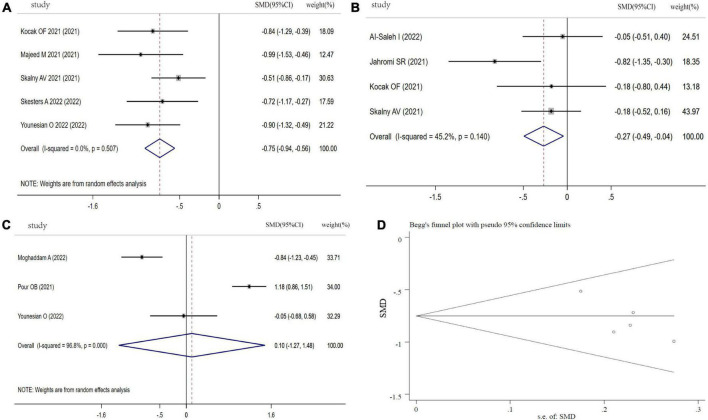
Forest plot of Se levels. **(A)** Se levels between COVID-19 patients and controls; **(B)** Se levels between severity status and non-severity status in COVID-19 patients; **(C)** Se levels between non-survivors and survivors in COVID-19 patients; **(D)** funnel plot of the meta-analysis on Se levels between COVID-19 patients and controls.

Figure 6A showed the results of 5 studies comparing the Se levels between COVID-19 patients and controls. From this forest plot, COVID-19 patients had significantly decreased Se levels in comparison with controls (SMD: −0.75, 95% CI: −0.94 to −0.56, *P* < 0.001, heterogeneity: *I^2^* = 0%, *P* = 0.52). Moreover, the pooled SMD of Se levels from 4 studies was significantly different between severity status and non-severity status (SMD: −0.27, 95% CI: −0.49 to −0.04, *P* = 0.02, heterogeneity: *I^2^* = 45.2%, *P* = 0.140, Figure 6B). However, three studies focused on Se levels between non-survivors and survivors in COVID-19, and Se levels in non-survivors was not different with survivors (SMD: 0.10, 95% CI: −1.27 to 1.48, *P* = 0.882, heterogeneity: *I^2^* = 96.8%, *P* < 0.001, Figure 6C). Sensitivity analysis indicated that the pooled SMD was not altered when any single study was excluded (Supplementary Figure 5). Funnel plot, Begg’s (*P* = 0.462) and Egger tests (*P* = 0.082) revealed no significant publication bias (Figure 6D).

## Discussion

COVID-19 has been a global pandemic for more than two years, and already have a great impact on our life quality and daily lifestyle. In view of the absence of specific medicine and effective vaccine, host immune function and nutritional status have been accepted the key factors in defending against SARS-CoV-2 attack. Among the nutritional factors, trace elements have been demonstrated to be involved in the modulation of immune responses, and their deficiencies act as the factors strongly affecting the course of COVID-19 disease (6). To our knowledge, this is the first meta-analysis to evaluate the levels of trace elements in COVID-19 patients, and explore the associations of trace elements and COVID-19 severity and poor outcomes. Our results support the potential clinical roles of Zn, Fe, Se in the evaluation of COVID-19 patients. Specially, circulating levels of Zn, Fe, and Se were significantly correlated with COVID-19 severity, and non-survivors in COVID-19 diseases displayed much lower Fe levels than survivors. This also reinforces the call for a specific nutritional management of COVID-19 patients.

Zn, as the second most abundant trace element in human body, depends on the daily food intake to achieve and maintain the adequate level of human tissues (73). The main functions of Zn include structural component, catalytic action, and regulatory function (74). Zn homeostasis is essential for anti-inflammatory, anti-redox reaction, development and differentiation of immune cells (such as T cell, B cell, natural killer cell, dendritic cell, and mast cell), and key cytokine release (such as IL-2, IL-6, and TNF-α) (75, 76). Previous studies have demonstrated Zn was involved in the overall functioning of human immune system, affecting innate immunity and adaptive immunity (77, 78). In addition, Zn also plays an important role in pathogens’ survival and the propagation of virulence(79). Rnai I, et al. indicated that nutritional intervention with Zn provided an adjutant therapy by eliciting their virucidal effects in the process of SARS-CoV-2 infection (80). Zn not only disrupted the balance of immune response, but also affected the expression of ACE2 receptors, which are required for SARS-CoV-2 entry into target cells (81). Moreover, Jennifer A Frontera, et al. reported that Zn was associated with increased rates of recovery and reduced risk of mortality among COVID-19 patients (82). A retrospective study showed high prevalence of Zn deficiency was positively associated with the COVID-19 cases/1 million populations in Asian countries (83). However, some studies showed controversial results on the relationship between Zn levels and COVID-19 infection and poor outcomes. Our meta-analysis result found that Zn level in COVID-19 patients was significantly lower than controls, and correlated with disease severity status.

Fe is a critical element involved in a variety of physiological functions, such as DNA biosynthesis, ATP generation, oxygen transport and storage, ROS production, energy production, and host defense (84). Disruption of Fe homeostasis is tightly associated with infection, cancer, cardiovascular disease, renal disease, and hematological disease(85, 86). Ferroptosis, a term coined in 2012, is an iron-dependent cell death pathway driven by excessive lipid peroxidation, which has been implicated in the development and disease of various tissues and organisms(87). Moreover, alteration of Fe distribution in COVID-19 patients is hypoferremia, and low Fe levels may impair hypoxia sensing and immunity (88). Hal et al. highlighted the evidence that Fe deficiency limited adaptive immunity and responses to vaccines (22). Therefore, The European Hematology Association (EHA) states that the populations should correct the Fe deficiency before administration of the COVID-19 vaccine. However, contradictory opinion suggested that the virus lead to the release of Fe from porphyrins by attacking and destroying hemoglobin, and the consequential result was the discharge of more Fe into the blood (89). Our pooled results showed that Fe levels in COVID-19 patients, severity status, and non-survivors were significantly lower than controls, non-severity status, and survivors, respectively.

Se is an indispensable trace element, necessary for human innate and acquired immunity, antibody production, muscle function, and signaling transduction pathways (90). Amounting evidence had demonstrated that Se deficiency was linked to higher susceptibility to RNA viral infection and poor outcome (91). Zhang et al. found an association between Se status and cure rate in COVID-19 patients in China (92). Alexander et al. made a full literature search and concluded that adequate supply of Se is essential for resistance to viral infections, immune function, and reduced inflammation, and mitigate the course of COVID-19 (32). However, Sobczyk et al. adopted a Mendelian Randomization (MR) analysis, and did not found the supplementation with Zn, Se, and Cu could prevent SARS-CoV-2 infection, critical illness or hospitalization for COVID-19 (93). A recent study also indicated that Se status or Se intake had no effect on humoral immune after vaccination (94). Our result showed that Se level in COVID-19 patients was significantly lower than controls, and the COVID-19 patients with severe status had lower Se level in comparison with non-severity status.

Cu plays an essential role in immune function and antioxidant defense (14). It is well-documented that Cu deficiency was associated with exceptional susceptibility to varied viral infections (95). The underlying mechanism by which Cu causes inaction of pathogens was still elusive (96). Mg, an essential substance, participates in many kinds of biochemical reactions, and also has anti-inflammation and anti-oxidant function. A nationwide retrospective cohort study including 1,150 counties, 287,326,503 individuals, and 5,401,483 COVID-19 confirmed cases revealed that the infection risk of the populations was distributed in low-magnesium areas in COVID-19’s early transmission (97). Tang et al. suggested that Mg supplementation may be a supportive treatment in COVID-19 patients (98). However, Cu and Mg, showed no difference between COVID-19 patients and controls, and had no relationship with disease severity and mortality in our meta-analysis. The possible reason is that the sample size is relative small, or the Mg and Cu levels in blood sample were not representatives of total Mg and Cu levels in body.

This study has several limitations. First, given the limited number of published literature, more evidence was needed to confirm the final results, especially for Mg, Cu, and Se. Second, it is possible that some undefined, or unreported factors might have contributed to the study heterogeneity. We did not conduct the subgroup analysis because of small samples. Third, some studies were retrospective studies, and the measurement methods were not described at all (e.g., laboratory assay, medical records, or self-reported). Forth, in view of study factors might not be independent of each other, we should perform the multivariable meta-regression. Having said that, it is possible that our results were substantially influenced by residential location, dietary habits, comorbidity, and population age. But, the majority of the retrieved articles were lack of full clinical information. Finally, publication bias was apparent in the comparison of Mg levels between COVID-19 patients and controls.

## Conclusion

In conclusion, our meta-analysis showed that COVID-19 patients had lower circulating Zn, Fe, and Se levels than healthy controls, and their levels were associated with the presence of severity status. Moreover, circulating Fe levels may provide part of the explanation for the unfavorable survival status. We strongly recommend that future studies make efforts to conduct a larger, multi-regional, and representative patient samples in order to uncover the roles of trace elements. Despite some doubts in the benefits of the trace elements, we presumed optimistically that supplements of trace elements might provide an adjutant treatment in the early stages of COVID-19.

## Data availability statement

The raw data supporting the conclusions of this article will be made available by the authors, without undue reservation.

## Author contributions

BL and YL conceived and designed the experiments and collected literature. YL and WL analyzed the data. BL wrote the drafts of the manuscript. YL checked and revised the manuscript. All authors interpreted the data and reviewed and approved the final version of the manuscript.

## Supplementary material

Supplementary Figure 1 Sensitivity analysis of Zn levels between COVID-19 patients and controls.

Supplementary Figure 2 Sensitivity analysis of Fe levels between COVID-19 patients and controls.

Supplementary Figure 3 Sensitivity analysis of Cu levels between COVID-19 patients and controls.

Supplementary Figure 4 Sensitivity analysis of Mg levels between COVID-19 patients and controls.

Supplementary Figure 5 Sensitivity analysis of Se levels between COVID-19 patients and controls.
