# Supplementary material for: Circulating trace elements status in COVID-19 disease: A meta-analysis
Source: Front Nutr. 2022 Aug 12;9:982032. doi: 10.3389/fnut.2022.982032 (PMC9411985; doi:10.3389/fnut.2022.982032)
Supplement: Supplementary Figure 1 — Sensitivity analysis of Zn levels between COVID-19 patients and controls. [file Data_Sheet_1.ZIP › supplemmentary materials/Table S1.docx]

**Table S1. Newcastle-Ottawa Scale assessment of included studies**

| **Author** | **Year** | **Country** | **Is the case definition adequate?** | **Representativeness of the cases** | **Selection of Controls** | **Definition of Controls** | **Comparability of cases and controls on the basis of the design or analysis** | **Ascertainment of exposure** | **Same method of ascertainment for cases and controls** | **Non-Response rate** | **Score** |
| --- | --- | --- | --- | --- | --- | --- | --- | --- | --- | --- | --- |
| **Included studies analyzing the difference between COVID-19 patients and controls** | | | | | | | | | | | |
| Nedic O | 2022 | Serbia | ★ | ★ |  | ★ | ★★ | ★ | ★ | ★ | 8 |
| Skalny AV | 2021 | Russia | ★ | ★ |  |  | ★ | ★ | ★ | ★ | 6 |
| Kocak OF | 2021 | Turkey | ★ |  |  |  | ★★ | ★ | ★ | ★ | 6 |
| PVSN KK | 2022 | India | ★ | ★ |  |  |  | ★ | ★ | ★ | 5 |
| Ivanova ID | 2022 | Bulgaria | ★ | ★ | ★ | ★ |  | ★ | ★ | ★ | 7 |
| Verschelden G | 2021 | Belgium | ★ | ★ |  |  |  | ★ | ★ | ★ | 5 |
| Jothimani D | 2020 | India | ★ | ★ |  | ★ | ★★ | ★ | ★ | ★ | 8 |
| Golabi S | 2021 | Iran | ★ | ★ | ★ |  | ★★ | ★ | ★ | ★ | 8 |
| Maares M | 2022 | Germany | ★ | ★ |  |  | ★ |  | ★ | ★ | 5 |
| Ghanei E | 2022 | Iran | ★ | ★ |  | ★ | ★★ | ★ | ★ | ★ | 8 |
| Elham AS | 2021 | Iran | ★ | ★ |  | ★ | ★★ | ★ | ★ | ★ | 8 |
| Majeed M | 2021 | India | ★ | ★ |  | ★ |  | ★ | ★ | ★ | 6 |
| Skesters A | 2022 | Latvia | ★ | ★ |  |  |  |  | ★ | ★ | 4 |
| Younesian O | 2022 | Iran | ★ | ★ |  |  | ★★ | ★ | ★ | ★ | 7 |
| Al-Jassas HK | 2022 | Iraq | ★ | ★ |  | ★ | ★★ | ★ | ★ | ★ | 8 |
| Bastin A | 2021 | Iran | ★ | ★ |  |  | ★★ | ★ | ★ | ★ | 7 |
| Yagci S | 2020 | Turkey | ★ | ★ | ★ | ★ | ★★ | ★ | ★ | ★ | 9 |
| **Included studies analyzing the difference between severity status and non-severity in COVID-19 patients** | | | | | | | | | | | |
| Jahromi SR | 2021 | Iran | ★ | ★ |  | ★ | ★ | ★ | ★ | ★ | 7 |
| Beigmohammadi MT | 2021 | Iran | ★ | ★ |  | ★ | ★★ | ★ | ★ | ★ | 8 |
| Skalny AV | 2021 | Russia | ★ | ★ |  | ★ |  | ★ | ★ | ★ | 6 |
| Zeng HL | 2021 | China | ★ | ★ |  | ★ |  | ★ | ★ | ★ | 6 |
| Kocak OF | 2021 | Turkey | ★ | ★ |  | ★ |  | ★ | ★ | ★ | 6 |
| AI-Saleh | 2022 | Saudi Arabia | ★ | ★ |  | ★ | ★ | ★ | ★ | ★ | 7 |
| PVSN KK | 2022 | India | ★ | ★ |  | ★ | ★★ | ★ | ★ | ★ | 8 |
| Yasui Y | 2020 | Japan | ★ | ★ |  | ★ | ★ | ★ | ★ | ★ | 7 |
| Zhu LY | 2021 | China | ★ | ★ |  | ★ | ★ | ★ | ★ | ★ | 7 |
| Quilliot D | 2020 | France | ★ | ★ |  | ★ |  | ★ | ★ | ★ | 6 |
| Bastin A | 2021 | Iran | ★ | ★ |  | ★ | ★ | ★ | ★ | ★ | 7 |
| Yagci S | 2020 | Turkey | ★ | ★ |  | ★ | ★ | ★ | ★ | ★ | 7 |
| Kilercik M | 2021 | Germany | ★ | ★ |  | ★ | ★★ | ★ | ★ | ★ | 8 |
| Tojo K | 2021 | Japan | ★ | ★ |  | ★ |  | ★ | ★ | ★ | 6 |
| Chakurkar V | 2021 | India | ★ | ★ |  | ★ | ★ | ★ | ★ | ★ | 7 |
| LV YL | 2021 | China | ★ | ★ |  | ★ | ★ | ★ | ★ | ★ | 7 |
| Lanser L | 2021 | Austria | ★ | ★ |  | ★ |  | ★ | ★ | ★ | 6 |
| Sonnweber T | 2020 | Austria | ★ | ★ |  | ★ |  | ★ | ★ | ★ | 6 |
| Zhao K | 2020 | China | ★ | ★ |  | ★ | ★★ | ★ | ★ | ★ | 8 |
| **Included studies analyzing the difference between survivors and non-survivors in COVID-19 patients** | | | | | | | | | | | |
| Zeng HL | 2021 | China | ★ | ★ |  | ★ | ★ | ★ | ★ | ★ | 7 |
| Pour OB | 2021 | Iran | ★ | ★ |  | ★ | ★ | ★ | ★ | ★ | 7 |
| Maares M | 2022 | Germany | ★ | ★ |  | ★ |  | ★ | ★ | ★ | 6 |
| Joulaei H | 2022 | Iran | ★ | ★ |  | ★ | ★ | ★ | ★ | ★ | 7 |
| Younesian O | 2022 | Iran | ★ | ★ |  | ★ |  | ★ | ★ | ★ | 6 |
| Moghaddam A | 2020 | Germany | ★ | ★ |  | ★ | ★ | ★ | ★ | ★ | 7 |
| Bonakdaran S | 2022 | Iran | ★ | ★ |  | ★ | ★ | ★ | ★ | ★ | 7 |
| Zhu LY | 2021 | China | ★ | ★ |  | ★ | ★ | ★ | ★ | ★ | 7 |
| Gunay S | 2021 | Turkey | ★ | ★ |  | ★ |  | ★ | ★ | ★ | 6 |
| Alamdari NM | 2020 | Iran | ★ | ★ |  | ★ | ★ | ★ | ★ | ★ | 7 |
| Yagci S | 2020 | Turkey | ★ | ★ |  | ★ | ★★ | ★ | ★ | ★ | 8 |
| Chakurkar V | 2021 | India | ★ | ★ |  | ★ |  | ★ | ★ | ★ | 6 |
| Lanser L | 2021 | Austria | ★ | ★ |  | ★ |  | ★ | ★ | ★ | 6 |
| Zhao K | 2020 | China | ★ | ★ |  | ★ |  | ★ | ★ | ★ | 6 |
| Nai A | 2020 | Italy | ★ | ★ |  | ★ | ★ | ★ | ★ | ★ | 7 |
| Hackler J | 2021 | Germany | ★ | ★ |  | ★ | ★ | ★ | ★ | ★ | 7 |
