# Supplementary figures and images for: Circulating trace elements status in COVID-19 disease: A meta-analysis
Source: Front Nutr. 2022 Aug 12;9:982032. doi: 10.3389/fnut.2022.982032 (PMC9411985; doi:10.3389/fnut.2022.982032)

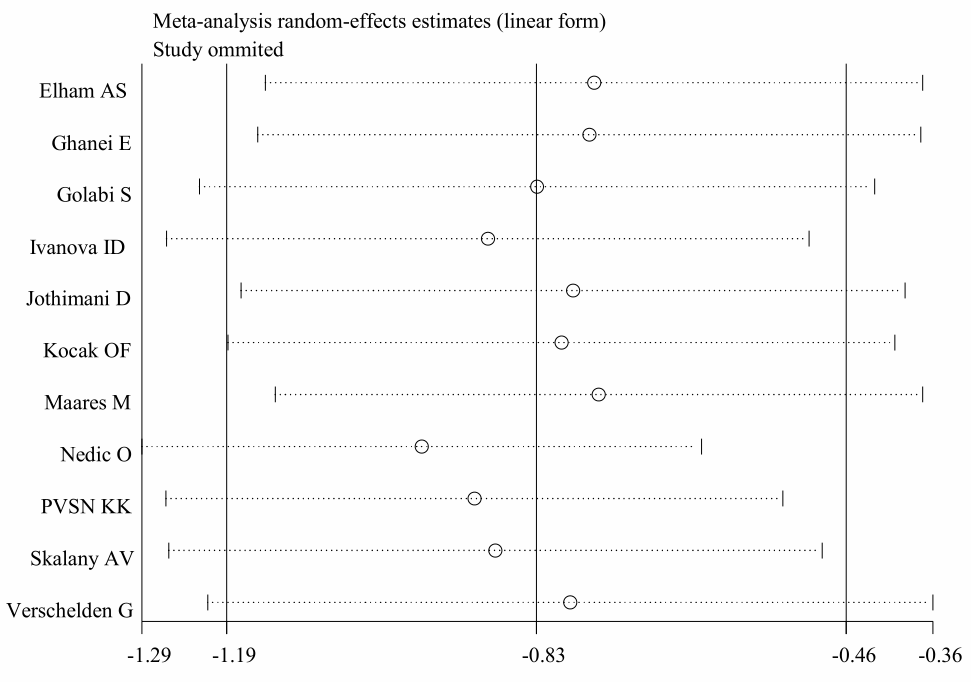

Supplement: Supplementary Figure 1 — Sensitivity analysis of Zn levels between COVID-19 patients and controls. [file Data_Sheet_1.ZIP › supplemmentary materials/Figure S1.tif]

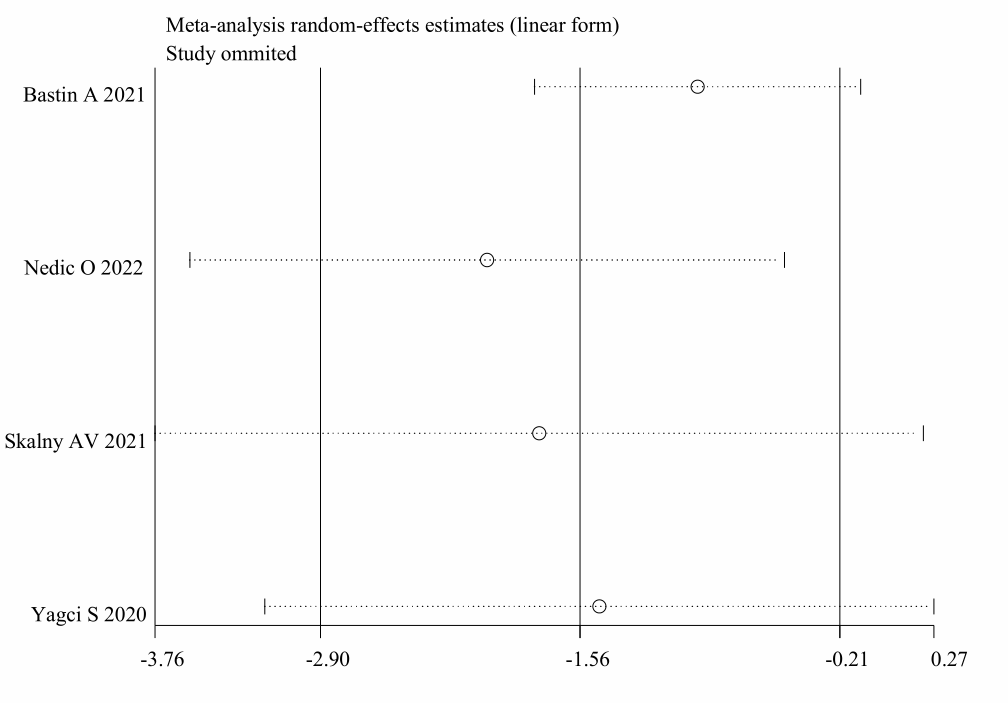

Supplement: Supplementary Figure 1 — Sensitivity analysis of Zn levels between COVID-19 patients and controls. [file Data_Sheet_1.ZIP › supplemmentary materials/Figure S2.tif]

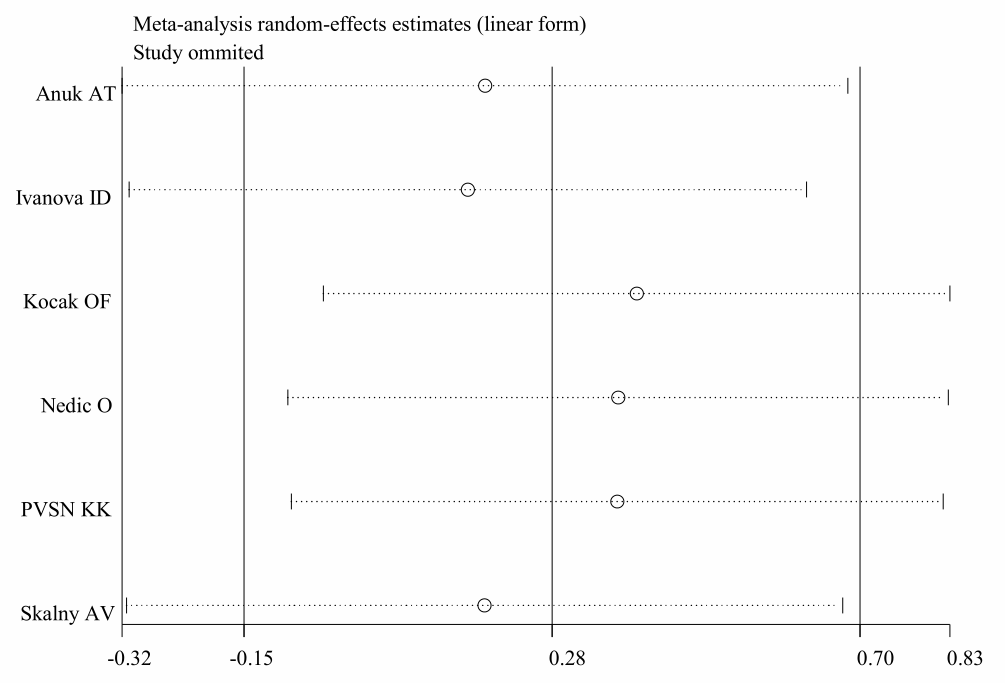

Supplement: Supplementary Figure 1 — Sensitivity analysis of Zn levels between COVID-19 patients and controls. [file Data_Sheet_1.ZIP › supplemmentary materials/Figure S3.tif]

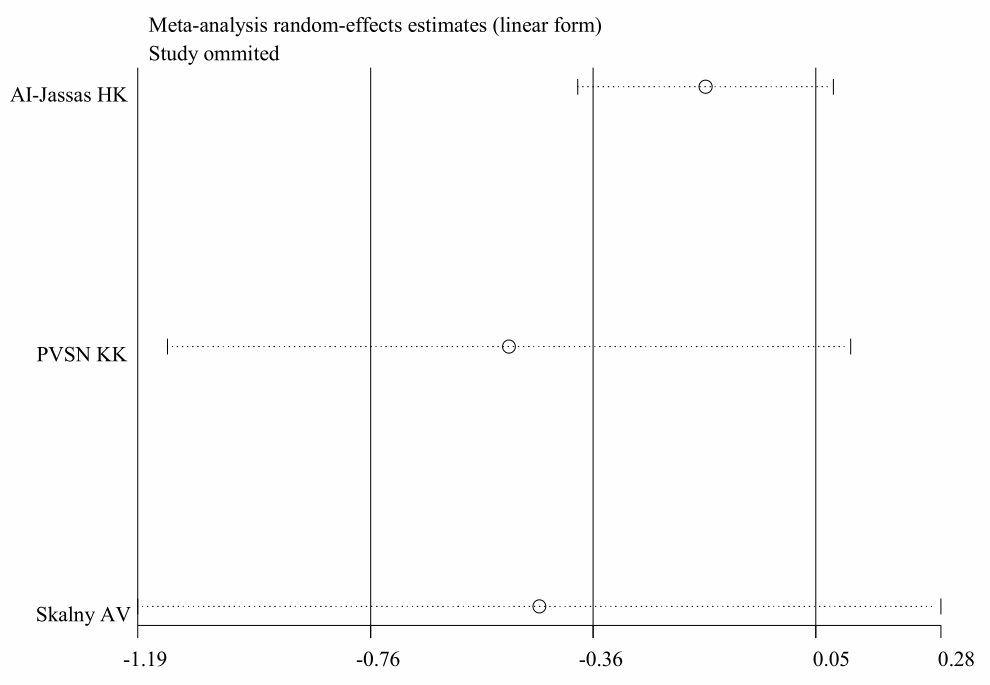

Supplement: Supplementary Figure 1 — Sensitivity analysis of Zn levels between COVID-19 patients and controls. [file Data_Sheet_1.ZIP › supplemmentary materials/Figure S4.tif]

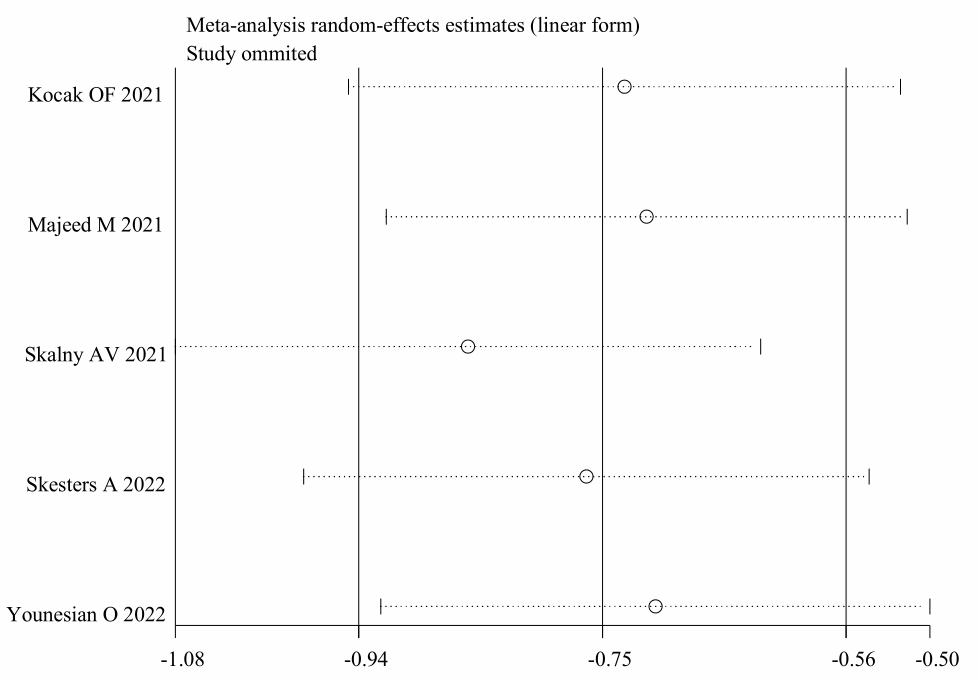

Supplement: Supplementary Figure 1 — Sensitivity analysis of Zn levels between COVID-19 patients and controls. [file Data_Sheet_1.ZIP › supplemmentary materials/Figure S5.tif]
